# Therapeutic Inhibition of the Complement System in Diseases of the Central Nervous System

**DOI:** 10.3389/fimmu.2019.00362

**Published:** 2019-03-04

**Authors:** Sarah M. Carpanini, Megan Torvell, Bryan Paul Morgan

**Affiliations:** ^1^UK Dementia Research Institute, Cardiff University, Cardiff, United Kingdom; ^2^Division of Infection and Immunity, School of Medicine, Systems Immunity Research Institute, Cardiff University, Cardiff, United Kingdom

**Keywords:** complement, therapeutics, CNS, neurodegeneration, injury

## Abstract

The complement system plays critical roles in development, homeostasis, and regeneration in the central nervous system (CNS) throughout life; however, complement dysregulation in the CNS can lead to damage and disease. Complement proteins, regulators, and receptors are widely expressed throughout the CNS and, in many cases, are upregulated in disease. Genetic and epidemiological studies, cerebrospinal fluid (CSF) and plasma biomarker measurements and pathological analysis of post-mortem tissues have all implicated complement in multiple CNS diseases including multiple sclerosis (MS), neuromyelitis optica (NMO), neurotrauma, stroke, amyotrophic lateral sclerosis (ALS), Alzheimer's disease (AD), Parkinson's disease (PD), and Huntington's disease (HD). Given this body of evidence implicating complement in diverse brain diseases, manipulating complement in the brain is an attractive prospect; however, the blood-brain barrier (BBB), critical to protect the brain from potentially harmful agents in the circulation, is also impermeable to current complement-targeting therapeutics, making drug design much more challenging. For example, antibody therapeutics administered systemically are essentially excluded from the brain. Recent protocols have utilized “Trojan horse” techniques to transport therapeutics across the BBB or used osmotic shock or ultrasound to temporarily disrupt the BBB. Most research to date exploring the impact of complement inhibition on CNS diseases has been in animal models, and some of these studies have generated convincing data; for example, in models of MS, NMO, and stroke. There have been a few recent clinical trials of available anti-complement drugs in CNS diseases associated with BBB impairment, for example the use of the anti-C5 monoclonal antibody (mAb) eculizumab in NMO, but for most CNS diseases there have been no human trials of anti-complement therapies. Here we will review the evidence implicating complement in diverse CNS disorders, from acute, such as traumatic brain or spine injury, to chronic, including demyelinating, neuroinflammatory, and neurodegenerative diseases. We will discuss the particular problems of drug access into the CNS and explore ways in which anti-complement therapies might be tailored for CNS disease.

## Introduction

### The Central Nervous System (CNS) as a Distinct Environment

**Figure 1:**
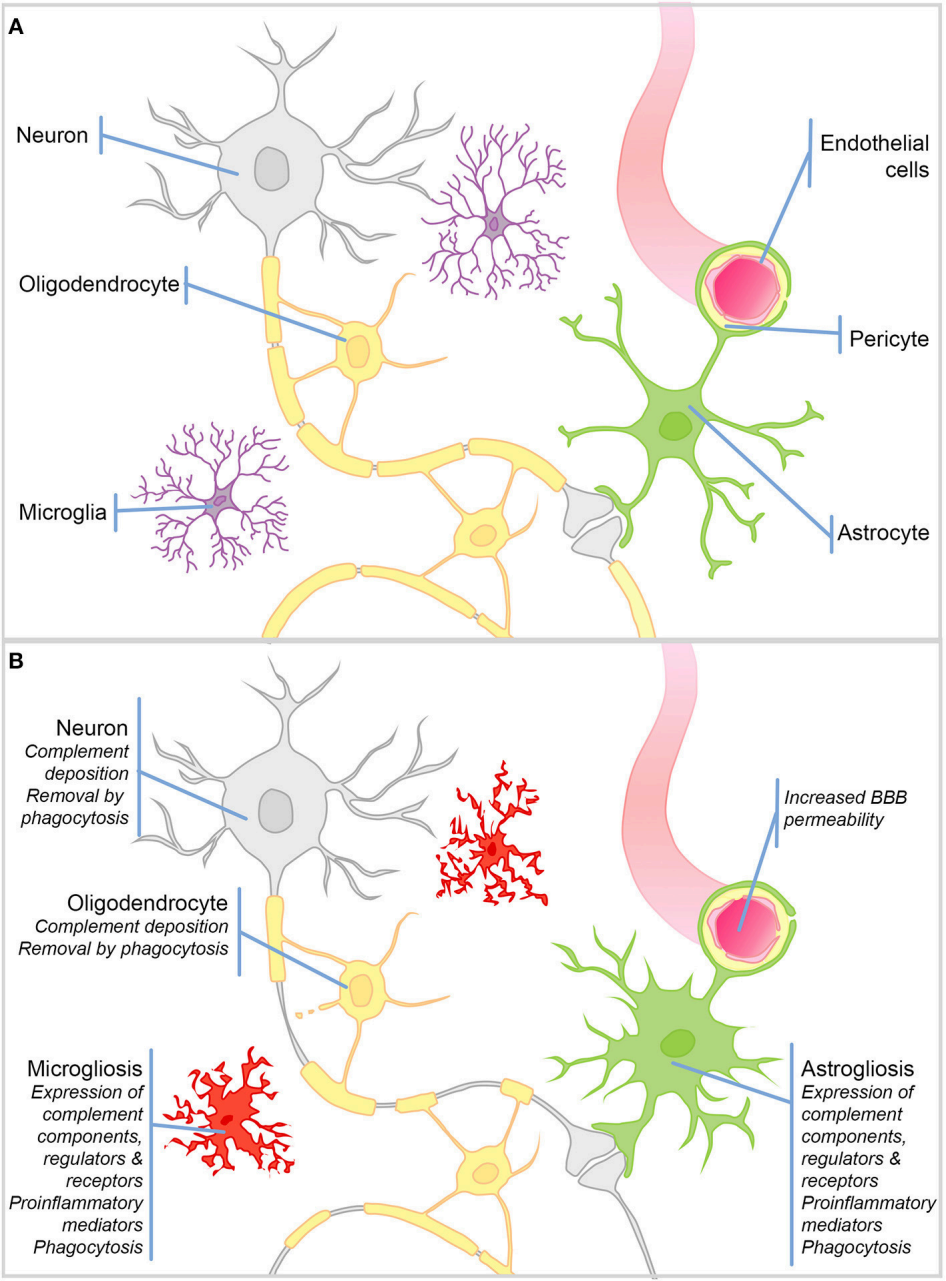
Schematic representation of cell types in the brain and their responses to injury. **(A)** Schematic representation of the cell types in the healthy brain. **(B)** During CNS injury and disease the BBB is compromised. There is significant microgliosis and astrogliosis, characterized by glial cell proliferation, upregulation of complement components, regulators and receptors, proinflammatory mediators, and active phagocytosis. Complement protein expression/deposition are increased on neurons and oligodendrocytes tagging them for removal by phagocytosis and driving neurodegeneration and demyelination.

The CNS was, for a long time, considered an immunologically privileged organ because the brain and spinal cord are protected from circulating inflammagens by the BBB. The BBB is a specialized membrane comprised of endothelial cells with tight junctions, vascular pericytes and perivascular glia (Figure 1A), which cooperate to form a selectively permeable barrier, protecting the CNS from fluctuating ion concentrations and circulating neurotransmitters, macromolecules, large proteins such as complement, and pathogens (1). However, isolation of the CNS is not absolute and there are a number of pathways by which systemic inflammation can be communicated to the CNS [reviewed; (2)]. Indeed, the recent demonstration of a CNS lymphatic system further undermines the concept of brain immunological privilege (3).

The healthy BBB forms early in development and restricts the infiltration of circulating immune cells into the brain parenchyma; hence, the dominant immune cells of the brain are the resident macrophage population—microglia (Figure 1B). This self-renewing (4), yolk sac-derived population develops within the CNS (5–7) and differs in many respects from macrophage populations found in the periphery (8–10). Compared to tissue macrophages, microglia are relatively “immune suppressed” due to expression of receptors for soluble signals in the extracellular milieu, for example, β2 adrenergic receptor binding of noradrenaline (11, 12), and signals delivered through direct contact with surrounding neurons, including CD200R, CX3CR1 (13, 14). The downstream signaling of such receptors suppresses the production of proinflammatory mediators and encourages a neuroprotective microglial phenotype. Resting microglia are relatively sessile, ramified cells; their numerous highly motile protrusions sample the entire brain every few hours (15, 16). Upon stimulation, these protrusions are withdrawn to create ameboid microglia that are migratory and upregulate expression of proinflammatory mediators and activating receptors involved in pattern recognition and phagocytosis (Figure 1B). This activated phenotype, if not kept in check, can cause havoc in the vulnerable CNS. More recently, it has been recognized that there are brain region-specific subpopulations of microglia with different responses to triggers and varying degrees of immune-vigilance (17, 18). There also exist resident non-microglial populations in the healthy brain including perivascular, meningeal and choroid plexus macrophages, which are capable of responding to noxious stimuli. In addition, during pathology blood borne macrophages and other immune cells are recruited to the injured brain as a result of increased BBB permeability. Astrocytes are a neglected cell type, despite the fact that they comprise ~70% of the cells in the brain, where they form syncytial networks around neurons. During health their primary role is homeostatic; they provide neurons with energy and neurotrophic support, and buffer ion and neurotransmitter concentrations [Reviewed elsewhere by Sofroniew and Vinters (19)—Figure 1A]. During inflammation, astrocytes demonstrate their immune-competence; they produce proinflammatory cytokines, are capable of phagocytosis, and can even present antigens to adaptive immune cells; however, acquisition of these immune roles is often associated with loss of homeostatic functions [(19)—Figure 1B].

Importantly for the subject matter of this review, there is compelling evidence that, during inflammation, not only microglia and astrocytes, but also neurons, oligodendrocytes, and endothelial cells in the brain, can express complement components, receptors, and regulators.

### The Complement System

**Figure 2:**
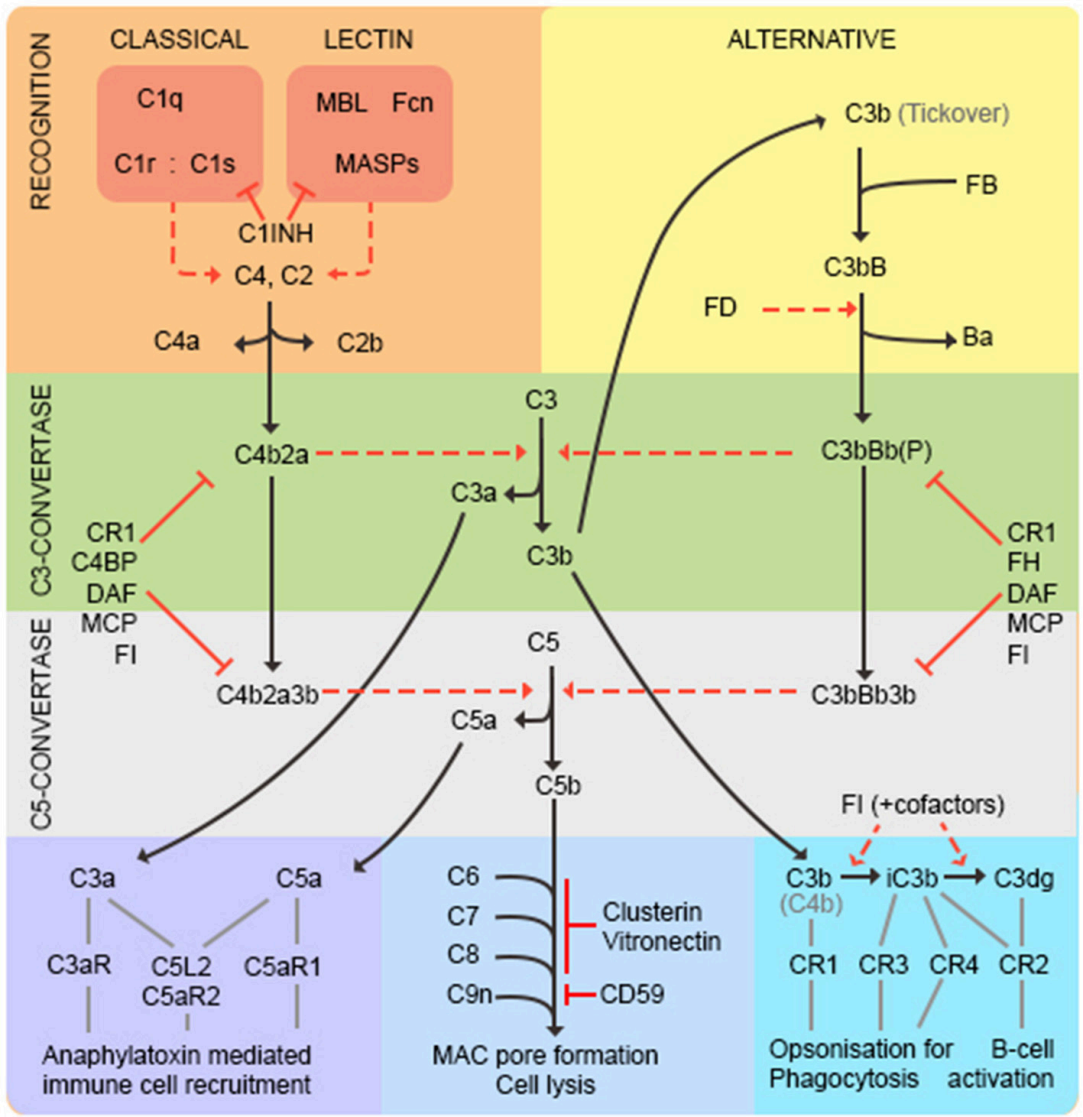
The complement pathway. The classical pathway is activated through antibody/antigen recognition by C1q in complex with C1r and C1s. The proteases C1r and C1s cleave C4 and C2 to generate the C3 convertase C4b2a regulated by complement receptor 1 (CR1), C4 binding protein (C4BP), decay accelerating factor (DAF), membrane cofactor protein (MCP), and factor I (FI). The lectin pathway is triggered by binding of mannose-binding lectin (MBL) or ficolins (FCN) to carbohydrate epitopes on targets. The MBL-associated serine proteases (MASPs) then cleave C4 and C2 to generate the C3-convertase as in the classical pathway. C1-inhibitor (C1INH) functions as a regulator to prevent excessive activation of both classical and lectin pathways. The alternative pathway is better considered as an amplification loop. C3b binds factor B (FB) to form C3bB. FB is cleaved by Factor D (FD) to form the C3bBb C3-convertase stabilized by properdin (P). This process is regulated by CR1, FI, factor H (FH), DAF and MCP. At this point the pathways converge—both C3-convertases cleave C3 to generate the anaphylatoxin C3a, and more C3b that binds to form the C5-convertases (C4b2a3b and C3bBb3b) that cleave C5 into C5a and C5b. C3a and C5a are potent anaphylatoxins that act through their respective receptors (C3aR, C5aR1, C5L2, and C5aR2) to recruit immune cells. C5b binds C6, C7, C8 (inhibited by vitronectin and clusterin) and multiple copies of C9 (inhibited by CD59) to form the lytic membrane attack complex (MAC). C3b opsonizes targets for phagocytosis and B-cell activation; C3b decays to iC3b then C3dg catalyzed by FI in the presence of cofactors (CR1, MCP, FH, C4BP).

Complement is recognized as an important branch of the innate immune system, providing the first line of defense against microorganisms. As complement is the subject of this issue, we will confine ourselves to a brief summary (represented in Figure 2). Complement comprises multiple recognition molecules that detect and bind target surfaces and recruit a cascade of protease enzymes and substrates, resulting in: (1) production of potent anaphylatoxins that attract and activate phagocytes; (2) formation of the lytic membrane attack complex (MAC); (3) target opsonization for phagocytosis and destruction (Figure 2). Three activation pathways, classical, lectin and alternative, converge on a common final pathway. The classical pathway is initiated by the C1 complex binding to antibody/antigen aggregates; the lectin pathway is triggered by binding of mannose-binding lectin (MBL) or ficolins to carbohydrate epitopes on targets; the alternative pathway is better considered as an amplification loop that is engaged regardless of the initial trigger. The activation pathways converge at the central C3 and C5 convertases, which generate potent anaphylatoxins C3a and C5a, C3b to opsonize surfaces facilitating phagocytosis and C5b to initiate MAC formation. The complement pathway mediates many of its effects through specific receptors on cells and is tightly controlled by regulators present on cells and in plasma, as discussed below (20).

The majority of complement proteins are predominantly synthesized in the liver (21, 22); however, it is becoming increasingly clear that complement proteins and their cognate receptors and regulators are expressed throughout the CNS. Most studies to date have utilized primary brain cell cultures and relevant cell lines and have identified complement expression at messenger RNA (mRNA) and/or protein level. Human primary oligodendrocytes expressed mRNA for all the components of the classical and terminal pathways and protein was detected for most of these (23). Human oligodendrocyte cells (HOG cell line) expressed the membrane complement regulators CD59, decay accelerating factor (DAF) and membrane cofactor protein (MCP) and secreted C1-inhibitor (C1INH), Vitronectin and Clusterin, whereas human astrocyte-derived cell lines expressed the same membrane regulators and the important C3/C5 fragment receptors complement receptor 1 (CR1) and C5a receptor (C5aR) (24, 25). Cultured microglia from human post-mortem brain (normal and Shy-Drager's syndrome) constitutively expressed mRNA for C1qB and C3 while C4 was expressed upon interferon (IFN)-γ stimulation (26). C4, C9, C1q, FH, C1INH, C3, C6, and Factor B (FB) were expressed in human neuronal cells *in vitro* whereas primary rat cerebellar granule cells expressed mRNA for C4, C1q, and C3 upon differentiation (27). Additionally, complement expression can be upregulated in disease; for example, C3, C1r and C1s expression was increased in primary microglia and astrocyte cultures from post-mortem brain upon exposure to cytokines associated with AD (28). Expression of C1INH, C1s, C1q and C3 mRNA was detected in AD and control brain extracts (29). Additionally, C1q, C3 and C4 gene expression was reported in primary microglia from AD patients (30). Cumulatively, the evidence suggests that nearly all complement proteins, regulators, and receptors are expressed in the CNS, and many are upregulated by inflammatory signals; it is therefore likely that a functional complement system is present in the CNS independent of peripheral complement.

### Roles of Complement in CNS Development

Complement proteins are involved in both prenatal and postnatal development of the healthy brain. In Xenopus embryos, morpholino knockdown of C3a receptor (C3aR) or blocking antibody against C3a administered during neural tube formation cause loss of neural crest cell organization, demonstrating a role for C3a and its receptor in the migration of neural crest cells (31). Central lectin pathway components Mannan-binding lectin associated serine protease (MASP)-1 and MASP-2 are highly expressed in the developing mouse brain with MASP (and C3) knockout mice showing defects in neuronal migration suggesting critical roles for complement activation in CNS development (32).

Complement also plays key roles in postnatal brain development. In humans and rodents, removal of redundant connections by synaptic pruning during childhood and early adult life is crucial for optimal brain function (33, 34). Using the developing rodent visual system as a model for synaptic pruning, it was shown that C1q and C3 (likely C3b/iC3b) localize to, and tag, specific synapses in the dorsal lateral geniculate nucleus (dLGN) for removal during development (35). C3 deficient (^−/−^) mice had improved hippocampal-dependent learning and memory (36), and failed to show the age-associated synapse loss observed in wild type animals (37), suggesting that complement is detrimental to synapse health. However, in a finding illustrating the dual nature of complement, C1q^−/−^, C3^−/−^, and C4^−/−^ mice all showed defects in synaptic pruning in the CNS that, in the former, associated with increased susceptibility to epilepsy (35, 38, 39). Furthermore, the C3b/iC3b receptor complement receptor 3 (CR3) is expressed on the surface of microglia and CR3^−/−^ mice showed defects in microglial engulfment of synapses, suggesting a collaboration between complement and microglia in synapse elimination (40). Taken together, these data highlight a critical involvement of the classical pathway in refinement of synapse networks during normal development.

### Complement and Neuroinflammation in CNS Disorders—Identifying Druggable Targets

As has been thoroughly reviewed elsewhere (41), inflammation in general and neuroinflammation in particular, is a double-edged sword, evolved to fight infection and restore or maintain homeostasis but, when uncontrolled, capable of wreaking havoc. The aim is therefore not to stop inflammation but encourage a protective rather than destructive profile and prompt resolution of inflammation. Given the important roles of complement in the developing brain, in defense against infection and in maintaining homeostasis, there may be situations where enhancing complement activity may be of benefit; however, in the context of CNS pathology, complement dysregulation leading to over-activation, has deleterious consequences and contributes to neuroinflammation.

Thus, the complement system offers an attractive drug target for these diseases that are currently without effective therapies. Drugging the complement pathway in the periphery is well researched and this knowledge could potentially be extrapolated to the CNS.

CNS disorders can be divided into acute, for example traumatic injury and stroke, and chronic, for example, demyelinating and neurodegenerative disorders, dependent on the causation, severity and duration. In the sections below, we will present evidence for the role of complement in both acute and chronic neurological disorders. We will not include stroke because this is discussed elsewhere in this issue and will also omit detailed discussion of the demyelinating diseases MS and NMO for brevity and because these have been well reviewed elsewhere (42, 43). Our overall aim is to provide insight into how complement therapeutics might impact these problematic diseases.

## Complement Proteins in Acute Neurological Disorders—Traumatic Brain and Spinal Cord Injury

### Traumatic Brain Injury (TBI)

TBI is classified as an injury to the brain due to trauma to the head via an external force; this can occur as a result of road traffic accidents, falls, sporting injuries or assaults; consequently, TBI is the major cause of brain injury and death in young adults in the Western world. TBI can cause diffuse or focal damage to the brain tissue and blood vessels depending on the type of injury. Subsequent to this primary injury, the BBB becomes compromised and there is a huge influx of cells, inflammatory mediators and plasma proteins, including complement proteins, that drive the delayed secondary inflammation, which is the major determinant of clinical outcome and thus recovery and survival (44). Human post-mortem TBI studies have shown increased expression of C3 and FB in brain and CSF (45) and both axonal and astrocytic expression of Clusterin (46). Increased soluble C5b-9 (terminal complement complex; TCC) levels were found in CSF after TBI, positively correlating with the degree of BBB damage (47), and further increased in response to secondary insults (oxygen deprivation/seizures) (48).

A wide array of TBI models are utilized in animal research, including cryoinjury, controlled cortical impact and standardized weight drop [models are reviewed elsewhere; (44)]. Increased C3 deposition, Clusterin and MAC deposition were observed alongside increased microglial and astrocytic activation markers after cortical contusion in the rat (49). Serum proteomics in Sprague-Dawley rats after “severe” deep cortical impact reported increased C9 and FB within the first few days after TBI (50). Complement deficient mouse models have been used to identify the impact of complement on neuropathology after TBI (Table 1). C3^−/−^ and C5^−/−^ mice showed reduced neutrophil extravasation upon traumatic brain cryoinjury (51); C4^−/−^ mice, but not C3^−/−^ or C1q^−/−^ mice showed reduced motor deficits and tissue damage following controlled cortical impact (52). In the same TBI model, CR2/CR1^−/−^ mice showed improved outcome with decreased mortality, neuronal cell death, C3 deposition, astrogliosis, and microgliosis (53). FB^−/−^ mice also showed reduced cell death in TBI with increased anti-apoptotic and decreased pro-apoptotic markers (54). In one study, MBL^−/−^ mice were protected from neurological injury following TBI (70); in contrast, another reported that MBL^−/−^ mice showed increased levels of degenerating neurons in the hippocampus CA3 region and impaired performance in non-spatial learning tasks (71). Despite several such inconsistencies, the studies to date suggest that deficiencies of individual complement proteins of the classical, alternative or terminal pathway improves outcome after TBI. However, all these studies used rodent models where the relevant protein is knocked out systemically from embryogenesis. Thus, it is important to identify whether anti-complement therapeutics administered immediately post-injury can have a similar beneficial effect, and to define the “therapeutic window of opportunity” for intervention post-injury.

**Table 1:** Consequence of complement deficiency on outcome of neurodegenerative disease.

	**Model**	**Deficiency**	**Consequence**	**Reference**
TBI	Traumatic brain cryoinjury	C3	Reduced pathology	(51)
	Controlled cortical impact	C3	No effect	(52)
	Controlled cortical impact	C4	Improved function	(52)
	Traumatic brain cryoinjury	C5	Reduced pathology	(51)
	Closed head injury	CR2	Improved function	(53)
	Controlled cortical impact	C1q	No effect	(52)
	Closed head injury	FB	Reduced pathology	(54)
SCI	T9 contusion	C1q	Improved function	(55)
	Contusion induced injury	FB	Improved function	(56)
	Weight drop	C3	Improved function	(57)
	Contusion injury	C5a	Improved function	(58)
	Contusion induced injury	CD59	Impaired recovery, increased injury	(56)
AD	Tg2576	C1q	Ameliorates synapse loss	(59)
	oAβ injection	C1q	Ameliorates synapse loss	(60)
	APP/PS1	C3	Ameliorates synapse loss	(60)
	J20 APP	C3	Exacerbated pathology	(61)
	oAβ injection	CR3	Ameliorates synapse loss	(60)
	APP/PS1	C3	Improved function	(62)
ALS	SOD1^G37R^	C1q	No effect	(63)
	SOD1^G37R^	C3	No effect	(63)
	SOD1^G37R^	C4	No effect	(64)
	SOD1^G37R^	C5aR1	Extended survival	(65)
HD	R6/2	C3	No effect	(66)
PD	MPTP induction of PD	C3	No effect	(67)
	MPTP induction of PD	C1q	No effect	(68)
	Paraquat/maneb induction of PD	CR3	Reduced dopaminergic neurodegeneration	(69)

*TBI, Traumatic brain injury; SCI, spinal cord injury; AD, Alzheimer's disease; ALS, Amyotrophic lateral sclerosis; PD, Parkinson's disease; HD, Huntington's disease*.

The majority of therapeutic studies in TBI models have focused on targeting the C3 convertase as a central player of all three activation pathways (Table 2). Administration of soluble CR1 (sCR1) pre- and post-TBI in rats reduced neutrophil accumulation in the injured brain (72). Administration of vaccinia virus complement control protein (VCP), an inhibitor of C3 activation, improved performance in spatial memory tasks after TBI (77). In mice, systemic inhibition of C3 via administration of Crry-Ig (recombinant chimeric Complement receptor 1-related protein Y (Crry) fused to mouse IgG1 Fc) 1 and 24 h post-TBI ameliorated neuronal damage in hippocampus and improved neurological outcome (87). Transgenic mice expressing soluble Crry (sCrry) specifically in astrocytes were protected in closed head injury TBI, with reduced C3 deposition, decreased BBB damage and improved neurological scores (88). Intravenous (iv) administration of C1INH 10 min post TBI reduced cognitive deficits and brain lesion size (89) and, in a separate study, improved motor scores, reduced cognitive dysfunction and reduced injury volume (90). Alternative pathway inhibition with systemically administered anti-FB reduced neuronal damage after TBI in mice (73). Lectin pathway inhibition using a multivalent MBL ligand improved functional and pathological outcome measures in a mouse TBI model (76). Terminal pathway inhibition using either the tick-derived C5 inhibitor OmCI or C6 antisense oligonucleotide decreased neuropathology and promoted recovery in severe closed head injury (74), and targeted inhibition of the terminal pathway using a CD59-CRIg hybrid that localized to areas of C3b/iC3b deposition in the injured brain was strongly neuroprotective in the same model (75). Recently, to determine which component or pathway of complement should be targeted for most efficient protection in TBI, three hybrid proteins, all containing CR2 to target to areas of complement activation, CR2-CD59 (inhibition of MAC), CR2-Crry (all complement pathways) and CR2-FH (alternative pathway), were compared; the latter two were most effective, demonstrating important roles for early activation products, both opsonins and anaphylatoxins (91).

**Table 2:** Consequence of pharmacological complement inhibition on outcome of neurodegenerative disease.

	**Model**	**Drug**	**Timing**	**Consequence**	**References**
TBI	Weight drop	sCR1	2 h and 2 min prior and 2 h post	Decreased neutrophil accumulation	(72)
	Weight drop	Anti-FB	1 and 24 h post	Decreased tissue damage	(73)
	Closed head	OmCI	Immediately prior, 15 and 30 min post	Improved function, reduced pathology	(74)
	Closed head	C6 α-sense oligoNT	6 days prior for 4 days	Improved function	(74)
	Cryoinjury	AcF	Immediately prior	Decreased neutrophil extravasation	(51)
	Closed head	CD59-2a-CRIg	30 min and 24 h post	Improved function	(75)
	Cortical impact	Polyman9	10 min post	Improved function, reduced pathology	(76)
	Lateral fluid percussion	VCP	15 min post	Improved function but not neuropathology	(77)
SCI	Weight drop	sCR1	1 h post and daily	Reduced degeneration	(78)
	Mild impact	VCP	Immediately post	Improved function and reduced pathology	(79)
	Pneumatic impact	C1inh	2 h post injury	Improved function	(80)
	Contusion	Anti-FB	1 and 12 h post injury	Improved function	(56)
	Compression	PMX53	45 min pre and 24 h post	Improved function	(58)
	Contusion	PMX205	14 days post	Detrimental for functional recovery	(81)
	Weight drop	CR2-Crry	1 h post	Improved function	(57)
AD	Tg2576, 3xTg	PMX205	After plaques for 2–3 mo 2x weekly	Reduction in fAβ deposits and activated glia	(82)
	APP/TTA	SB290157	3x week for 5 weeks from 7.25 mo	Reduction in Aβ deposits	(83)
	Oligo Aβ	ANX-M1	17 and 2 min pre and 24 and 48 h post	Prevented synapse loss and impairment of LTP	(60)
ALS	SOD1^G93A^ rat	PMX205	P28 and P70	Improved function	(84)
	hSOD1^G93A^ ms	PMX205	P35 (pre) and P31 (post)	Improved function	(85)
HD	3-NP rats	PMX53	2 days prior	Improved function	(86)
	3-NP rats	PMX205	2 days prior and 2 days post	Improved function	(86)

*TBI, Traumatic brain injury; SCI, spinal cord injury; AD, Alzheimer's disease; ALS, Amyotrophic lateral sclerosis; PD, Parkinson's disease; HD, Huntington's disease*.

### Spinal Cord Injury (SCI)

SCI can be caused by sudden traumatic insults that crush or sever the cord, or non-traumatic injuries, for instance, triggered by cancer, arthritis or infection and usually compressing sections of cord; here we will restrict discussion to traumatic causes. SCI results in dysfunction and sometimes complete loss of function below the lesion site. Symptoms are often life-long and, since SCI is most common in under-30s, is associated with huge personal and health-care costs (https://www.spinal-research.org/). In traumatic SCI the primary pathology is caused by a mechanical force directly damaging the neural tissue—this primary insult is difficult to protect against. However, post-injury inflammation, with infiltration of immune cells and production of pro-inflammatory mediators, results in secondary pathology in adjacent areas characterized by oedema, ischemia, and excitotoxicity (92). There is considerable blood-spinal cord barrier (BSCB) damage and resultant inflammation in this secondary phase; despite this, studies of complement in SCI are scarce. Early human studies showed elevated C3, C4, and C5 levels in plasma of patients post-SCI suggestive of an acute phase response (93). As with TBI, rodent models of SCI vary widely; many different approaches to inducing injury have been taken, including weight drop, contusion, compression, laceration, and chemical injection. Complement proteins were deposited at sites of SCI in rodents; C1q, FB, C4, and TCC expression all increased at and around the injury within 24-h post-SCI and remained high up to 6 weeks (94, 95), and FH and Clusterin were elevated in lesioned neurons and oligodendrocytes (96). In a less severe weight-drop contusion SCI mouse model, C3 (likely C3b/iC3b) was deposited in white matter at the site of injury at 4-h and more widely in adjacent white and grey matter at 12- and 24-h, returning to baseline by 3 days post-injury (57).

C3^−/−^ mice were significantly protected in contusion-induced SCI with reduced lesion size, necrosis, demyelination, and neutrophil infiltration, improved locomotor score and accelerated recovery (57); C1q^−/−^ mice showed decreased lesion volume and improvements in locomotion and fine motor control compared to controls in the same model (55). Mice deficient in FB subjected to contusion SCI showed accelerated recovery of locomotion, marked improvements in macroscopic tissue integrity, and reduced demyelination, C3 and C9 deposition and infiltration of neutrophils and macrophages compared to controls (56), while mice deficient in the terminal pathway inhibitor CD59 showed increased pathology with loss of myelin structure, scarring and vacuolation, hemorrhage, neutrophil and macrophage infiltration, and TCC deposition (56). C5aR^−/−^ mice showed acute but not long-term improvements in functional recovery (97). *In vitro* studies showed that C1q-treatment increased cortical neurite length on myelin by inhibition of growth cone repulsion by myelin associated glycoprotein (MAG); however, comparison of C1q KO and C1q WT mice in a peripheral conditioning lesion model of SCI showed no differences in axon length, lesion volume or scarring, although C1q deficiency was associated with increased axonal turning (98).

There are currently no proven therapies for SCI, unsurprising given the many obstacles in promoting re-wiring of axons and remyelination. Preventing or reducing the inflammation-driven secondary phase offers opportunity; indeed, methylprednisolone is the only currently available treatment though its effectiveness is unclear (99–101). There have been a few studies of anti-complement agents in SCI rodent models and the majority of these have utilized iv administration, possible because of BSCB disruption post-injury. Injection of the C3 convertase inhibitor VCP into the injured spinal cord in a rat SCI model, restored spinal cord tissue integrity, reduced macrophage and microglial activation and improved acute motor deficits (79, 102); iv administration of recombinant sCR1 in mice 1 h post-SCI and daily thereafter reduced neuron swelling, degeneration, necrosis and neutrophil infiltration and improved recovery (78); iv C1INH 2-h post-SCI in rats improved motor recovery, reduced lesion volume and leukocyte infiltration (80). Alternative pathway inhibition with iv anti-FB mAb accelerated recovery and reduced lesion size in the same model (56). In a contusion SCI mouse model, iv administered CR2-Crry localized to the lesion site, improved locomotor deficits and reduced necrosis, demyelination, and neutrophil infiltration (57); because CR2-Crry targets specifically areas of pathology, it is bioavailable in the SCI when given at a dose that does not influence circulating complement activity, reducing the risk of infections and other undesirable effects of systemic complement inhibition. Another strategy to reduce infection risk is to target C5a, a potent chemoattractant, or its receptor C5aR1 (CD88), a G protein-coupled receptor (GPCR) expressed on granulocytes monocytes/macrophages peripherally and on astrocytes and microglia (and at low level neurons and oligodendrocytes) in CNS (103, 104). Two small cyclic peptide C5aR1 antagonists PMX53 and PMX205 (86, 105) have been tested in SCI models; iv administration of PMX53 improved functional recovery, and reduced macrophage/microglial numbers, expression of pro-inflammatory cytokines IL-1β and TNF-α and astrogliosis in mice compared with controls (58). In a rat SCI model, the impact of PMX205 administration was dependent on timing post-injury and was linked to the sequence of immune cell recruitment to the site; whereas early administration accelerated recovery, late administration inhibited the macrophage/microglial response and slowed functional recovery and re-myelination following injury, further emphasizing the importance of timing interventions (81). Mice treated with C5aR antagonist [hydrocinnamate—(OpdChaWR)] showed acute but not long term improvements in functional recovery (97). Additionally, bone marrow chimeric mice lacking peripheral but not central C5aR showed no differences from control mice. Together these data suggest an initially detrimental role of C5aR followed by a delayed neuroprotective role, likely mediated by CNS resident cells.

Taken together, these studies indicate that inhibition of classical/lectin and/or alternative pathways or specific effectors like C5a can be efficacious in SCI. Timing of interventions may be crucial to avoid impacting beneficial clearance roles of complement. Terminal pathway inhibition has not been tested in the models but the impact of CD59 deficiency noted above suggests that this is a viable target.

## Complement Proteins in Chronic Neurological Disorders—Demyelination and Neurodegeneration

Inflammation was noted early as a feature of chronic brain disease; indeed, astrogliosis and microgliosis were included in the original descriptions of AD by Alois Alzheimer over a century ago. Despite this, a classification divide emerged with diseases like MS considered inflammatory while diseases like AD were considered degenerative. The artificial nature of this divide has become clear in recent years with the realization that there are many shared features. The evidence implicating inflammation as a driver of pathology in chronic neurodegenerative diseases is now substantial and includes genetic studies identifying inflammatory risk genes (106), clinical studies demonstrating that long-term treatment with non-steroidal anti-inflammatory drugs (NSAIDs) is protective in humans (107) and mouse models (108) and the observation that systemic infections and inflammation increase the risk and/or rate of progression of dementia (109–111). Complement, the focus of this review, goes hand-in-hand with inflammation and represents a potential driver of chronic CNS diseases.

### Alzheimer's Disease (AD)

AD is the leading cause of dementia affecting almost 50 million people worldwide, a number projected to increase to 150 million by 2050 (https://www.alz.co.uk/research/statistics). AD is characterized by two hallmark pathologies; amyloid-β (Aβ) plaques and neurofibrillary tangles comprising hyperphosphorylated tau. Recent studies have implicated complement in AD pathogenesis. Genome wide association studies identified single nucleotide polymorphisms (SNPs) associated with risk of late-onset AD in genes encoding complement proteins Clusterin (*CLU*) and CR1 (*CR1)* (106, 112, 113). Biomarker studies have identified complement proteins and activation products in plasma and/or CSF that distinguish AD from controls and predict risk of progression to AD (114–117). Immunohistochemistry (IHC) of post-mortem AD brain revealed complement proteins and activation products decorating plaques and tangles. In particular, classical pathway proteins C1q, C3, and C4 co-localized with amyloid fibrils, Aβ deposits and neurofibrillary tangles, notably in temporal cortex, amygdala, and hippocampus, in AD brain (118–120). The terminal pathway activation marker TCC was abundant in AD cortex in association with aggregated Aβ, neurofibrillary tangles and neuropil threads (121). Cells expressing C5a receptors C5aR1 and C5L2 were associated with neurofibrillary tangles, neuropil threads, and dystrophic neurites in AD plaques in hippocampus and frontal cortex (122). A weakness of these IHC studies is that they are performed on post-mortem brain, inevitably end-stage disease, and do not provide insight into early disease or disease progression. A large-scale microarray study of young, healthy old and AD brains identified marked changes in complement expression with ageing, and elevated expression of *C4A, C4B, C3aR1, C5aR1, CFHR1*, and *CLU* in AD compared to age-matched controls; C1q binding protein (C1qBP) expression decreased in AD (123). Increased C1q expression in brain with ageing (healthy or AD) has been robustly replicated (59, 124, 125).

Mechanisms of complement activation in the AD brain have been studied *in vitro* and in animal models. Aβ fibrils activate and consume complement classical and alternative pathways *in vitro* and generate C3a, C5a, and TCC (119, 126). C5a administration resulted in death of primary mouse neurons in culture; this could be blocked by addition of C5aR1 antagonist PMX53, demonstrating that C5a (acting via C5aR1) is sufficient to induce neuronal cell death *in vitro* (127). Animal models have underpinned the majority of research into roles and mechanisms of complement in AD. Most mouse models mimic the rare early-onset forms of AD in which single gene mutations have been identified rather than the common polygenic late onset AD, and thus individually mimic only certain aspects of the disease; it is therefore unsurprising that different models yield different and often contradictory results. Despite these reservations, these mouse models have aided understanding of AD pathology. Broadly, models can be divided into three groups: Aβ pathology; Tau pathology; both. These mouse models recapitulate many of the pathologies found in AD brain; for example, in the PS/APP model fibrillar Aβ plaques form and C1q localizes to these plaques (128). Back-crossing AD mouse models onto complement deficiencies has been used to determine the role of complement in the pathophysiology of AD (Table 1). Deficiency of C1q (classical pathway) in Tg2576 (Aβ pathology) mice reduced glial activation and synaptic loss without influencing Aβ load compared with controls (59). Genetic deletion of C1q, C3 or CR3, all of which are required for effective opsonization and phagocytosis of synapses, reduced microglial numbers and synapse loss when crossed to two different Aβ models (J20 and APP/PS1); further, when Aβ fibrils were injected directly into brain, C1q deficiency protected from toxicity (60). In contrast, C1q deficiency in the 3xTG model exacerbated neurodegeneration because of a loss of C1q-triggered expression of neuronal survival pathways (129). Others showed that C3 deficiency improved performance on learning and memory tests and decreased microglia and astrocyte number associated with plaques in an Aβ model (APP/PS1) (62); in contrast, C3 deficiency was associated with increased amyloid burden, decreased neuronal staining and activated glia in the J20 (Aβ) model (61). Despite the evidence noted above that Tau pathology is associated with complement, there was a dearth of studies in Tau models; two recent publications have changed this. Administration of blocking anti-C1q antibodies in a mouse Tau model (P301S) inhibited microglial synapse loss and rescued synapse density (130), while C3aR deletion attenuated neuroinflammation and reduced synaptic deficits and neurodegeneration in the PS19 Tau model (131).

As is clear from the anti-C1q experiment described above, mouse models also offer a way to test *in vivo* the impact of complement therapeutics on disease (Table 2). The C5aR1 antagonist PMX205 decreased amyloid and tau deposits, reduced activated glia and improved cognition in two Aβ models (Tg2576 and 3xTg) (82). Levels of C1q and C3 were unchanged upon PMX205 treatment, suggesting that their physiological functions are preserved. As noted above, blocking antibody against C1q [(ANX-M1/ANX005); Annexon Biosciences] protected from synapse loss in Aβ models and reduced toxicity of Aβ fibrils injected into the lateral ventricles (60); this agent showed no toxicity, even at high doses (200 mg/kg) and has proceeded to clinical trials (132). A note of caution in the use of anti-complement agents comes from a study of C3 inhibitor sCrry administered to an Aβ model (hAPP × TGFβ1) which resulted in increased Aβ deposition and neuronal degeneration (133).

The evidence—genetic, clinical, and from models—implicating complement as a driver of pathology in AD is compelling. A complicating factor is that complement may also have protective roles in clearing debris in early disease. Improved understanding of the time course of complement involvement may identify therapeutic windows where complement inhibitors will improve outcome.

### Amyotrophic Lateral Sclerosis (ALS)

ALS, also known as Lou Ghering's disease, is an adult onset neurodegenerative disease, usually fatal within 2–5 years of onset (134). ALS is caused by progressive loss of upper and lower (α) motor neurons (135), resulting in denervation of neuromuscular junctions in the peripheral nervous system, progressive muscle weakness, atrophy, spasticity, respiratory failure, and ultimately paralysis and death. Approximately 90% of ALS cases are sporadic and 10% familial. Causative missense point mutations have been identified in superoxide dismutase (*SOD1*), TAR DNA binding protein (*TDP-43*), fused-in-sarcoma-protein (*FUS*), and chromosome 9 open reading frame 72 (*C9orf72*). The only currently available treatment for ALS is Riluzole, an ion channel blocker and inhibitor of glutamate release which modestly increases survival (136, 137).

Neuroinflammation is a consistent feature of ALS with abundant reactive microglia and astrocytes and T-cell infiltration observed (138). IHC identified increased C1q protein in motor cortex and spinal cord of ALS post-mortem tissue; C3 activation fragments and TCC were also demonstrated in areas of pathology (139, 140). C3c labeled astrocyte-like cells in the former study whereas C1q and C3d co-localized with neurons, astrocytes and microglia, and TCC primarily microglia, in the latter. Others described C4d and TCC staining of degenerating neurons and glia in ALS motor cortex and spinal cord (141) and C5aR1 upregulation in areas of pathology (142). C3d and C4d were also found on oligodendroglia and degenerating neurites, surrounded by CR4-positive microglia, in spinal cord and motor cortex (141, 143). C1q, C3, and TCC were present on motor end-plates in intercostal muscles in ALS donors even early in the disease process (144); DAF and CD59 were upregulated at the end-plates, perhaps reflecting a response to complement activation and TCC/MAC deposition. TCC immunoreactivity at end-plates negatively correlated with α-bungarotoxin staining, implicating TCC/MAC in loss of end-plates (144). In myasthenia gravis, end-plate destruction is dependent on complement activation and MAC formation (145), supporting a causative role in ALS.

The source of complement in ALS pathology is unclear; the BBB is disrupted in the disease (146); however, local biosynthesis likely also contributes. *In situ* hybridization demonstrated upregulated *C1qb* and *CLU* mRNA in areas most affected by neurodegeneration (147); more recently, increased C1q and C4 expression by glial cells was demonstrated in ALS cord white matter (140) indicating a local source of complement. Complement expression positively associated with increased infiltration of dendritic cells and CD8+ T-lymphocytes from the periphery (140, 141). Biomarkers also implicate complement. Complement activation products C3c and C4d were present in CSF and correlated with disease severity scores (148–150). Levels of C5a and TCC were significantly elevated in ALS plasma, and leukocytes from ALS patients had increased surface (C5aR1-bound) C5a (151). These biomarker findings strongly support a role for complement dysregulation in ALS patients; however, the nature and location of complement protein deposition in different studies was contradictory, perhaps due to differences in disease stage or comorbidities.

Numerous rodent models of ALS have been generated based on known causative mutations in SOD1, responsible for ~10% of familial ALS. Rodents over-expressing human mutant SOD1^G93A^ recapitulate key neuropathological and functional hallmarks of ALS, characterized by lumbar motor neuron loss which correlates with progressive motor deficits and ultimately paralysis, and by inflammatory changes including robust astrogliosis, microgliosis, and BBB-disruption (152–154). Complement dysregulation is apparent from increased expression and deposition of C1q, C4, FB, C3 activation products and TCC, increased expression of C5aR1, and reduced expression of complement regulators DAF and CD59 (64, 84, 144, 154–156). Complement deposition has also been observed in sciatic nerves (64) and at the neuromuscular junction (156) in ALS models, consistent with the concept that complement contributes to nerve terminal destruction in ALS. In the TDP43^Q331K^ mouse model, progressive motor deficits, astrogliosis, and microgliosis correlated with complement dysregulation in the spinal cord; expression of C1qB, C4 and C3 was elevated and DAF mRNA reduced in the lumbar spinal cord and in tibialis anterior muscle of TDP43^Q331K^ mice compared with controls (157). Immunofluorescence confirmed markedly increased C1q and C5aR1 in motor neurons and microglia.

Surprisingly, C1q deletion in SOD1^G37R^ ALS mice exacerbated synaptic loss at end-stage and it was implied that this was a consequence of increased microglial phagocytosis; however, C1q deletion did not significantly affect disease onset, progression, or survival and had no effect on global astrogliosis, microgliosis, or neuronal loss (63) (Table 1). Deletion of the gene encoding C4, which is necessary for activation of both the classical and lectin pathways, significantly reduced the number of activated macrophages found in sciatic nerves of mSOD1^G93A^ mice but again failed to influence the disease course (64). C3 deletion also failed to affect overall survival or motor neuron loss in SOD1^G93A^ ALS mice (63); the finding that deletion of C3, central to all complement pathways, fails to rescue disease has provoked the suggestion that complement does not contribute to ALS disease progression (at least in this model). The demonstration that anti-complement drugs ameliorate disease in a similar model contradicts this suggestion (Table 2). Oral administration of C5aR1 antagonist PMX205, even when given in established disease, reduced weight loss and motor deficit scores, slowed disease progression and enhanced survival times in SOD1^G93A^ rats and mice (84, 85). These functional improvements were associated with reduced astrocyte proliferation, reduced influx of proinflammatory monocytes and granulocytes and an increase in the CD4+: CD8+ T-cell ratio, consistent with the reported neuroprotective role of CD4+-T cells in ALS (158). The same authors showed that deficiency of C5aR1 (upregulated in human and rodent ALS) extended survival in SOD1^G93A^ mice (65). Taken together, these data strongly implicate the C5a/C5aR1 axis in disease and identify it as a target for therapy in ALS.

### Huntington's Disease (HD)

HD is an autosomal dominant, inherited neurodegenerative disease characterized by progressive motor symptoms, psychiatric disturbances, and dementia. It is caused by expansion of a three-base-pair (CAG) repeat (39–121 repeats vs. normal range 8–39 repeats) in exon 1 of the *HTT* gene that translates into a polyglutamine tract at the N-terminus of the protein. This results in a polyglutamine length-dependent misfolding and accumulation of huntingtin protein in the striatum and cortex (layers 3, 5, and 6) followed by neuronal loss in these areas which spreads to the hippocampus (159, 160). Neuropathology of HD is graded based on Vonsattel staging (161) dependent on the severity of neuronal loss, astrogliosis, and brain atrophy. Precisely how the huntingtin trinucleotide expansions result in neuronal death and associated gliosis remain unclear. Microglial activation can be demonstrated by PET scanning even in early disease and correlates with disease severity (11C-raclopride binding) (162); indeed, even in pre-symptomatic gene carriers, microglial activation was present and correlated with striatal neuronal dysfunction and with risk of developing HD within 5 years (163).

HD post-mortem tissue showed abundant reactive astrogliosis and microgliosis and intranuclear ubiquitin positive inclusions in the caudate and temporal lobes (164). IHC showed that neurons, astrocytes and myelin sheaths in the HD caudate and striatum were immunoreactive for C1q, C4, C3 and neo-epitopes in iC3b and TCC (164). Expression of mRNA encoding early complement components C1q (c-chain), C1r, C3, and C4, complement regulators C1INH, Clusterin, MCP, DAF and CD59, and complement receptors C3a and C5a was upregulated in the HD striatum. Early disease stages did not stain for complement suggesting that early neuronal damage precedes local complement synthesis and activation. Microarray analysis in HD post-mortem tissue demonstrated increased expression of complement components C4A, C4B and C3, most significantly in the most affected areas, caudate nucleus, and motor cortex (165). Unbiased proteomic profiling revealed 18 proteins that were differentially expressed in HD plasma, several of which are involved in the innate immune system; Clusterin, C7 and C9 increased with disease severity (166).

Early animal models of HD utilized toxin-mediated striatal lesions; for example, Lewis rats given intracerebral 3-nitropropionic acid (3-NP), an inhibitor of the mitochondrial citric acid cycle, developed striatal lesions, weight loss, gait disturbances, dystonia and ataxia, thus reproducing some of the pathological and clinical characteristics of HD (86). Oral administration of C5aR antagonist (PMX53 or PMX205) reduced weight loss and motor deficits, even when given post-toxin administration, whereas NSAID, ibuprofen, and a TNF-α inhibitor (infliximab) had no significant functional impact, suggesting that ability to rescue these deficits hinged on the complement pathway *per se* rather than neuroinflammation in general (Table 2). 3-NP treatment caused lesions with robust neuronal death and neutrophil infiltration and surrounded by C5aR-, C3-, and C9-positive glia. C5aR blockade reduced lesion volume and number; lesions contained fewer apoptotic cells and astrocytes and were no longer surrounded by complement-positive glia. While these data were a helpful proof of concept (and this was the first paper demonstrating that PMX53 and PMX205 cross the BBB), the model used is extremely artificial, acute and invasive, unlike the chronic, cumulative dysfunction seen in HD.

R6/2 transgenic mice provide a more realistic HD model; these mice express exon 1 of the human *huntingtin* gene, including a pathological trinucleotide repeat; they develop progressive behavioral and neuropathological deficits, including synaptic loss, but do not develop neuronal loss and fail to demonstrate upregulation of complement proteins (66). It is, therefore, unsurprising that C3 deficiency did not alter disease progression in this model. C5aR was the only complement molecule upregulated in the model and it remains undetermined whether targeting the C5a-C5aR1 axis would be beneficial.

### Parkinson's Disease (PD)

PD is characterized by loss of dopaminergic neurons in the substantia nigra and deposits of the protein α-synuclein that form the pathological hallmarks of the disease, Lewy bodies. Patients present with resting tremor, bradykinesia, and rigidity. Complement activation has been associated with α-synuclein and Lewy bodies in Parkinson's disease; *in vitro* studies demonstrated that the disease-associated splice variant α-synuclein 112, but not the full length protein, cause activation of complement (167). *In vivo*, C3d, C4d, C7 and C9 localization in Lewy bodies was reported in one study (168), although this was not recapitulated in a separate study (169). More recently, deposition of iC3b and C9 in Lewy bodies and melanized neurons was reported; iC3b immunoreactivity increased with normal ageing and was further elevated in PD vs. age-matched controls (170). A correlation was described between the ratios of C3/Aβ42 or FH/Aβ42 in CSF and severity of Parkinson's disease motor and cognitive symptoms, but not with absolute levels of C3 or FH (171).

Although there are many mouse models of PD, drug or neurotoxin induced, or genetic, none fully replicates the human disease (172). A few studies have explored roles of complement in these models; absence of C3 in mice did not protect against depletion of dopaminergic neurons in the toxin-induced MPTP model (67) (Table 1). There was an increase of C1q in relevant brain regions in this model but C1q deficiency did not protect from disease (68). A very recent study identified a role for CR3 in activation of the microglial NADPH oxidase (Nox2) and subsequent neurodegeneration in a toxin-induced PD model; CR3 knockout mice were protected from dopaminergic neuron loss and motor dysfunction, suggesting that complement opsonization and CR3 engagement contribute to the disease process (69).

## Targeting Complement in Neurological Disease

### Getting Therapeutics Into the CNS

Having made the case above for an involvement of complement in acute neurological injuries and neurodegenerative diseases, attention naturally turns to therapeutic significance. There is a huge and growing complement therapeutics industry with a myriad of new drugs emerging; however, to date CNS targets have been largely ignored (173). Drug delivery is a major limiting factor for CNS therapies that needs to be considered when designing therapeutics for treating neurological conditions. The BBB precludes passive entry of molecules larger than ~400 kDa thus occluding entry of macromolecules, including antibody and protein therapeutics. In TBI and SCI the BBB is impaired to some degree, enabling drugs to access the injured areas (47). Treatment options to access the CNS in diseases where the BBB remains intact include both invasive and non-invasive techniques [reviewed in (174, 175)]. Historically, access of drugs to the CNS involved disruption or damage to the BBB or the use of pharmacological agents to increase its permeability; however, in many cases this resulted in widespread neuronal damage and an associated inflammatory response. Less damaging ways of opening the BBB include the use of focused ultrasound waves of low intensity that cause local and temporary disruption to the BBB and administration of “osmotic shock” agents (176). Pharma companies have designed a host of other strategies to deliver therapies, including Trojan horse delivery, use of viral vectors, nanoparticles and chimeric peptides, expanded on below.

Trojan horse technologies involve the creation of fusion proteins that lock the drug to a delivery component that utilizes receptors in the BBB, such as the insulin receptor and transferrin receptor, to enable bidirectional transport into and out of the brain. As an example of this concept, a recombinant anti-Aβ single chain fV antibody (fragment variable region only) fused to a fAb fragment of an anti-insulin receptor mAb bound the insulin receptor at the BBB, was transcytosed across the barrier enabling it to access and recognized Aβ within the brain and was then shuttled out again with its Aβ cargo for disposal (177). Anti-complement therapeutic antibodies, of which there are many in the clinic or in development, could be similarly piggy-backed into the brain to inhibit complement.

Small, hydrophobic molecules can cross the BBB via lipid-mediated diffusion. As an example, oral administration of the small molecule NLRP3 inhibitor MCC950 in PD mouse models reduced nigrostriatal dopaminergic degeneration, motor deficits and accumulation of α-synuclein through inhibiting inflammasome activation (178). Several small-molecule complement inhibitors are in development but, with the exception of the anti-C5aR1 molecules PMX53 and PMX205 described above, these have yet to be assessed for BBB permeability.

### Targeting Complement in CNS

Eculizumab, a humanized anti-C5 antibody, is the lead anti-complement drug but to date has only been approved for two rare disorders, paroxysmal nocturnal hemoglobinuria (PNH) and atypical hemolytic uremic syndrome (aHUS) and recently for Myasthenia Gravis. In a recent small trial of eculizumab in NMO, a demyelinating disease characterized by BBB disruption and inflammation/degeneration of the optic nerve and spinal cord (www.clinicaltrials.gov NCT00904826), treatment reduced the number of neurological episodes (179). This study has raised the prospect of using eculizumab for other inflammatory CNS diseases, although the BBB is likely a much greater hurdle in these other conditions and it is unclear whether they can be treated systemically. There is an urgent need to apply rational drug design for targeting complement activation in the CNS to obtain effective treatments with low side-effects and costs; for example, there is little point in designing an anti-C1q therapeutic to be administered systemically for a CNS disease given that C1q exists throughout the body and in the circulation at micromolar levels—extremely high drug doses would be required to have any impact at the desired site in the CNS even if the drug is BBB penetrant. Given the ubiquitous expression of complement proteins throughout the body and the role of complement proteins in fighting infection and maintaining homeostasis, anti-complement therapeutics at these doses would likely have consequences throughout the body. Rather, therapies should target areas of pathology, as described above for the fusion proteins linking CR2 (localizes to C3 activation products in tissues) with a complement regulator, or target complexes, for example MAC, which exist at much lower concentrations and are found only in areas of pathology.

A common stumbling block in designing treatments for neuroinflammatory disorders is timing. Despite early conceptions about the fixed nature of post-mitotic neurons in the CNS, there is a great deal of redundancy and flexibility in neuronal circuits. Networks are hence able to compensate for a huge amount of cell loss through synaptic plasticity so that, by the time patients present at the clinic with symptoms, major neuronal death has already occurred. Inflammation as a cause and consequence of this neuronal death occurs early and, unlike in acute conditions, fails to resolve because the primary stimulus, cell death, accumulation of toxic proteins or mitochondrial dysfunction, persists. Thus, effective treatments that aim to halt or slow disease progression must be administered early—likely before symptoms are apparent—and must prevent further neuronal cell death *and* encourage resolution of inflammation. Evidence from studies of the impact of NSAIDs on neurodegeneration support the idea that early and long-term treatment is protective but treatment post-onset fails (reviewed by 106). Early intervention requires ways of identifying those at high risk; genetic studies have identified polygenic signals that include many inflammatory genes and are highly predictive of risk of AD (106), and inflammatory biomarkers may also help predict risk (114). For large scale screening of pre-symptomatic populations expensive interventions such as brain imaging or CSF sampling are not practicable; in contrast, plasma offers an attractive source of biomarkers, although the levels of inflammatory markers in plasma may not reflect inflammation in the CNS. Simple and highly predictive plasma biomarkers are emerging and are likely to transform treatment of AD and other neurodegenerative diseases in the near future (180).

With regard to anti-complement therapies, it is likely that different approaches will be needed for different diseases; identifying for each disease when complement is activated, which pathways are activated and what the consequences are will be essential for effective interventions. Studies to date have been restricted to models and have focused on targeting the C3 convertases, central to all three activation pathways and thus a blunt tool likely to impact immune defense and other beneficial functions. The implication of C5a/C5aR in several CNS diseases offers the prospect of more targeted therapy with less risk of iatrogenic disease, although the systemic impact of long-term inhibition of the C5a/C5aR axis are uncertain. Evidence from models obtained either by complement gene deletion (Table 1) or anti-complement therapies (Table 2) has been helpful but are often contradictory, studies reporting both positive and negative impacts in similar models; this likely results from differences in timing and precise nature of the intervention and highlights once again the need for a thorough understanding of the underpinning biology prior to human studies.

## Concluding Remarks

Therapy of acute neurological injury and neurodegenerative diseases represent a major therapeutic challenge. Most of the diseases described above currently have no effective therapies and new approaches are desperately needed. Although there are some common features, notably inflammation and complement activation, the described diseases are very heterogeneous, even within disease labels—AD is not a single disease! Patient stratification, for example, selecting patients with high inflammatory markers and evidence of ongoing complement activation for anti-complement drug therapy, will be necessary for successful trails in the future; this requires better biomarkers. For most of the diseases, proof of concept for new approaches to therapy is stymied by the lack of good models; critically, agents that are effective in current models usually fail in human trails (https://www.nature.com/articles/d41586-018-05722-9). For AD, models that better reflect the human disease are now available and may help overcome this issue. Switching off complement systemically will impact immune defense; while this may not be an issue for short-term therapy in acute conditions, in chronic diseases requiring life-long treatment it is a major consideration. Despite all these problems, inflammation and complement activation present tractable targets in neuroinjury and neurodegenerative disease and deserve investment into basic understanding and the development of CNS-targeting anti-inflammatory and anti-complement drugs.

## Author Contributions

All authors listed have made a substantial, direct and intellectual contribution to the work, and approved it for publication.

### Conflict of Interest Statement

The authors declare that the research was conducted in the absence of any commercial or financial relationships that could be construed as a potential conflict of interest.
